# Selective vulnerabilities and biomarkers in neurocognitive aging

**DOI:** 10.12688/f1000research.10652.1

**Published:** 2017-04-13

**Authors:** Zachariah Reagh, Michael Yassa

**Affiliations:** 1 Department of Neurobiology and Behavior, Center for the Neurobiology of Learning and Memory, Institute for Memory Impairments and Neurological Disorders, University of California, Irvine, CA, USA

**Keywords:** neurocognitive aging, episodic memory, entorhinal cortex

## Abstract

As the world’s population continues to age, an understanding of the aging brain becomes increasingly crucial. This review focuses on several recent ideas and findings in the study of neurocognitive aging, specifically focusing on episodic memory, and discusses how they can be considered and used to guide us moving forward. Topics include dysfunction in neural circuits, the roles of neurogenesis and inhibitory signaling, vulnerability in the entorhinal cortex, individual differences, and comorbidities. These avenues of study provide a brief overview of promising themes in the field and together provide a snapshot of what we believe will be important emerging topics in selective vulnerabilities in the aging brain.

## Introduction

As of 2015, the global percentage of individuals 65 years of age or older was estimated to be 8.5%, which is projected to grow to 16.7% by 2050 ^1^. Increasing age is a primary risk factor of many disease states, including dementias such as Alzheimer’s disease (AD). Beyond disease risk, however, even ‘healthy’ aging significantly alters functional and structural properties of the brain. Furthermore, there is considerable individual variability in aged individuals in terms of both cognitive and neural measures ^2, 3^. Thus, it is imperative that we understand age-related changes to the brain in both the presence and absence of frank pathology such as that of dementing illnesses. Such an understanding will provide valuable insight into ‘healthy’ aging as well as potential biomarkers for differentiating this trajectory from diseases.

In this review, we will discuss important prevailing themes that can serve to guide our thinking about neurocognitive aging. We will also highlight some recent findings that may offer promising directions in the search for biomarkers and potential targets of intervention:

**1. Aging is a story of dysfunction rather than cell loss**

This first point is perhaps the least contentious and has been important in guiding our thinking about how aging affects cognition. Though the brain undergoes volumetric changes with age, this is thought to reflect synaptic degradation rather than frank neuronal loss in the absence of disease. Moreover, loss of function in particular neuronal circuits may be far more striking and informative at early stages of the aging process. It is worth noting that this understanding forms an important background that frames the other findings we discuss below.

**2. Neurogenesis is a complex biomarker**

Until very recently, it has been fairly well accepted that neurogenesis significantly declines in the aged brain, which may account for declines in memory ability. However, recent findings in the human brain have complicated this view.

**3. Letting go of your inhibition**

Many areas of the brain—notably, regions involved in memory—are typically under tight inhibition. This inhibitory control is dysregulated in aging, which can have important consequences on circuit-level computations.

**4. Entorhinal cortex: the gateway to neurodegeneration**

A wealth of evidence points to entorhinal cortex (EC) as being among the earliest brain regions affected by aging, and recent studies suggest that certain subdivisions of the EC may be especially vulnerable. In addition to the EC, several new candidate regions of vulnerability, including thalamic nuclei and the locus coeruleus, have emerged in recent years.

**5. Individual differences are informative**

Individuals vary widely in terms of both cognition and neurobiology as they age. We often collapse across and ignore many such differences in any given experiment, but they likely provide important clues about a person’s cognitive and clinical trajectories.

**6. It is time to embrace and understand comorbidities**

Brain aging is inexorably linked to other factors that influence the brain’s health as well as the rest of the body. However, these comorbidities are often viewed as ‘nuisance’ variables that we attempt to exclude. These are important factors that should be considered for a full and valid understanding of the aging brain.

## Aging is a story of dysfunction rather than cell loss

Neuronal loss in the medial temporal lobes (MTLs) is observed in histological studies of AD, which correlates with disease onset ^4, 5^. Although hippocampal volume is often found to decrease in ‘healthy’ aging ^6, 7^, there is little evidence to suggest that ‘healthy’ aging features comparable loss of cells ^8^. What could account for this discrepancy? It is likely that such volumetric changes are due to more subtle losses of cellular complexity, including degeneration of synapses ^9^ and axons ^10^. This is consistent with studies using unbiased stereology in animal models of aged rodents, all of which show that there is little to no evidence of frank cell loss in the aging brain ^8^. Additionally, in specific transgenic mouse models of AD, impairments in memory-guided tasks correlate more closely with synaptic dysfunction than neuronal loss ^11^. Though likely less drastic than cell death, these kinds of changes may contribute to subtle cognitive perturbations. For instance, the hallmark decline of episodic memory ability with aging may arise from highly specific synaptic loss observed in the perforant path between the EC and the hippocampus ^8^. In humans, white matter loss specific to this projection correlates with poorer mnemonic discrimination (disambiguating overlapping events in memory), thought to rely on hippocampal pattern separation ^12, 13^. In a typical mnemonic discrimination paradigm, participants encode a set of items and later are tasked with rejecting similar ‘lure’ items as being distinct from the studied set. Importantly, these discrimination paradigms have been shown to be highly sensitive to age-related decline in episodic memory ability.

Beyond morphological changes that do not correspond to neuronal loss per se, changes to the functional properties of brain systems have been reported that correlate with cognitive deficits. In the hippocampi of aged rats there are several functional changes that are associated with poorer memory performance. This includes alterations to excitatory synaptic transmission and synaptic plasticity across all hippocampal subfields ^14^. Several studies have also noted that elevated levels of activity in the CA3 subfield correlate with impaired mnemonic discrimination ^15, 16^, a finding mirrored in humans with high-resolution functional magnetic resonance imaging (fMRI) ^17^. Behaviorally, mnemonic discrimination paradigms are sensitive to subtle age-related deficits across multiple domains (for example, objects, space, and time) ^18– 20^. Other fMRI experiments examining activity during task-free rest report decreased correlations in activity among ‘default mode’ brain regions ^21^, many of which are strongly implicated in memory. Importantly, many of these studies control for gray matter volume in their analyses such that global atrophy does not explain these disrupted correlational patterns among brain regions. Together, these findings suggest that noninvasive functional approaches such as behavioral studies and measures of brain activity at rest or driven by mechanistically targeted tasks are potentially very fruitful avenues of inquiry.

## Neurogenesis is a complex biomarker

Though adult neurogenesis (the birth of new neurons in the mature brain) has been known for some time to occur in the dentate gyrus of the mammalian hippocampus ^22^, the phenomenon has recently become a popular avenue of research ^23^. In rodents, nearly 10,000 new cells are generated per day ^24^ and only half of these survive ^25^. Among these surviving granule cells, most integrate into existing hippocampal circuits and may replace existing granule cells ^26^. Though the precise functions of adult neurogenesis are not known, disruption of neurogenesis is detrimental to certain mnemonic processes, primarily pattern separation ^27^. It must be noted, however, that many memory-guided behaviors are unaffected by this. Adult neurogenesis reportedly decreases with age in the rodent brain ^28^, leading some to propose it as a mechanism for curbing age-related memory loss and detecting pathological states along different aging trajectories ^29^.

A recent study using nuclear bomb test–derived ^14^C in genomic DNA found that although hippocampal neurogenesis does decline with age in humans, the effect is nowhere near as drastic as in rodents ^30^. Moreover, after age 20, the loss of neurogenesis dropped at a low and fairly monotonic rate even into advanced age. Although this does not refute a role for neurogenesis in explaining human age-related memory decline, the relationship is unclear. It must be noted that there are differences between human and rodent adult neurogenesis. For instance, compared with rodents ^30^, a greater number of newborn neurons in the human dentate gyrus integrate into existing circuits ^31^. There is also evidence to suggest that the dynamics and behavioral relevance of neurogenesis differ between rats and mice ^32^. Nonetheless, targeting neurogenesis as a mechanism for therapeutic intervention has some promise, but further research is needed to fully understand its role in the human brain across the life span.

## Letting go of your inhibition

As previously noted, hyperactivity in hippocampal CA3 in aged animals is associated with diminished memory ^15, 16^. This likely stems at least in part from reduced inhibitory signaling from somatostatin-positive GABAergic interneurons to CA3 ^33^. Similarly, somatostatin-positive interneurons are selectively lost in the hilar region of the dentate gyrus in older animals with memory impairments ^34^. As hippocampal networks are typically under tight inhibition, such alterations can drastically affect mnemonic processes. For instance, elevated firing rates in the dentate gyrus may reduce the sparsity of firing in granule cells, thereby reducing its capacity for creating independent representations. The same change in the CA3 region may facilitate runaway excitation in recurrent collaterals and further interfere with the network’s ability to represent new information. This is likely to lead to failures in pattern separation and instead biases the hippocampus toward erroneous generalization ^35, 36^ or the generation of ‘false’ memories.

In a recent series of studies in rats ^37^, humans ^38^, and transgenic mice ^39^ with AD pathology, a low dose of the anti-epileptic drug levetiracetam was found to reduce hippocampal hyperactivity in memory-impaired older individuals as well as rescue cognitive deficits to normal levels. Hippocampal hyperactivity is therefore a strong candidate biomarker in neurocognitive aging that may differentiate ‘healthy’ from pathological aging. Moreover, although both the acute and long-term effects of the relevant drugs must be studied more extensively, targeting and reducing hyperactivity in specific brain networks provide a well-supported mechanistic avenue for treating cognitive decline.

It should be noted, however, that loss of inhibition is not universal across the aging brain but rather may be regionally specific. Some regions in fact exhibit *greater* inhibition in advanced age. For example, evidence in both primates ^40^ and rodents ^41^ associates aging with increased inhibitory tone in the prefrontal cortex. Thus, inhibitory control is a broad and potentially very important issue to consider.

## Entorhinal cortex: the gateway to neurodegeneration

Though the hippocampus is often studied in the context of dementia and is often predictive of AD ^42^, histological ^43^ and neuroimaging ^44^ studies have shown that the EC is among the earliest brain regions affected by AD-related pathology, such as cortical thinning and the accumulation of hyperphosphorylated tau protein. Recent evidence suggests that there may be detectable changes in the functional properties of EC. An elegant study by Khan and colleagues ^45^ demonstrated that, across mice and humans, metabolism in the lateral portion of the EC (LEC) was affected in preclinical AD, consistent with other studies in rodent models of aging ^46^. Moreover, positron emission tomography (PET) imaging of hyperphosphorylated tau deposition in the EC is a promising biomarker ^47^.

Critically, although it has been known for some time that LEC and medial EC (MEC) are functionally dissociable in rodents ^48, 49^, evidence in humans has only recently been demonstrated ^50– 52^ (though in humans the division may be more anterolateral versus posteromedial ^50, 51^). Generally, cells in MEC are thought to represent spatial configurations via grid-like firing patterns ^48^, whereas those in LEC are thought to represent objects or items within those configurations ^49^. Task-driven dissociations between LEC and MEC ^52^ provide a valuable opportunity to test key changes to the aging human brain. In particular, one might hypothesize that LEC shows selective functional impairments in aged adults, and the extent of this impairment might inform us as to one’s neurocognitive trajectory. A recent study by Olsen and colleagues provides evidence for such a view via structural changes to EC subdivisions ^53^. Future studies can further address this question and can resolve ambiguities in behavioral outcomes such as whether functions of the LEC are broadly impacted by ‘healthy’ aging ^54^ or only in the case of a clinical trajectory ^55^.

In addition to the EC, several other regions have recently surfaced as candidate locales for neurodegenerative changes in AD. The first is the locus coeruleus, which is a major brainstem hub for producing norepinephrine and which recently was identified as a region that accumulates tau pathology early in AD ^56^ and may even transfer this pathology to the EC via a prion-like mechanism ^57^. Second, there is evidence that the thalamus ^58^, striatum ^58^, and basal forebrain cholinergic system ^59^ are also compromised as regions expressing early pathology and neurodegenerative changes in AD. A recent study reported evidence that basal forebrain degeneration may actually precede and dictate AD-related pathology in the EC ^60^. Moving forward, it will be important to elucidate which regions exhibit neurodegenerative or volumetric change related to AD versus volumetric change related to synaptic and dendritic loss, which may be a part of ‘healthy’ aging.

## Individual differences are informative

Our use of quotations around ‘healthy’ aging here is quite intentional. It is well noted that brain aging is not a uniform process but rather consists of many different cognitive and neurobiological phenotypes ^61^. This raises an important question: what exactly constitutes a normal aging trajectory? Absent a disease state, cognitive abilities vary widely across older individuals ^2, 62^, and there is evidence suggesting that early life education ^63^ and genetic factors ^64^ influence outcomes. Moreover, individuals with a common diagnosis may share a wide array of distinct symptoms or pathological phenotypes. In many experimental studies, it is common to attempt to reduce or even eliminate as many variables as possible for a ‘cleaner’ examination of a variable of interest. This is often a matter of cost or practicality in a given study, as more variables may necessitate larger sample sizes. Nonetheless, we suggest that attempting to understand these differences and relate them to experimental outcomes is a necessary step in understanding neurocognitive aging more broadly.

Several studies across rodents ^65^, primates ^66, 67^, and humans ^18– 20^ have embraced individual differences in behavior, which sometimes can cleanly dissociate subgroups of aged individuals. In one novel and exciting approach, recent studies by Madan and Kensinger ^68, 69^ found that the structural complexity of certain brain regions (including MTL and striatum) is a more sensitive measure of age-related differences than volume and cortical thickness. This approach leverages individual variability rather than controlling for its effects as a set of confounding variables. If combined with extensive demographic information and sensitive behavioral data, a technique such as this might provide a powerful means of understanding factors that impact and arise from aging.

## It is time to embrace and understand comorbidities

Related to individual variability, comorbidities provide a considerable challenge to characterizing a normal neurocognitive aging process. As many as one third of individuals in the US 65 years or older are diagnosed with at least two chronic diseases ^70^. Prominently, aging itself is the primary risk factor for AD and shares pathological features such as amyloid deposition ^71^. One major factor is cerebrovascular infarction (stroke), which is fairly common to some extent in a large proportion of older individuals ^72^. In addition to major stroke events, more chronic forms of cerebrovascular disease such as microbleeds, or ‘silent infarcts’, have been found to affect cognition absent any AD-related pathology ^73^. Relatedly, recent evidence has pointed to a strong role of inflammation in many age-related diseases, including arthritis, diabetes, dementia, and metabolic dysregulation ^74^. Aging upregulates a number of pro-inflammatory signaling molecules, which may dysregulate immune responses in the nervous system with damaging effects ^75^. Diabetes and metabolic syndrome—an increasingly widespread issue encompassing obesity, hypertension, insulin resistance, and related symptoms—have also been linked to age-related cognitive decline and AD ^76^. Finally, differences in neuropsychiatric health exist among aged adults. For example, many individuals go on to develop late-life depression ^77^, which can affect not only cognition but core neurobiological processes as well.

As with individual differences, comorbidities are frequently treated as nuisance variables in a given study. Although there have been some efforts, a systematic understanding of comorbidities and how they can be leveraged to improve our grasp on the aging process has not been provided. One promising example comes from studies of the oldest old where the best indicator of the likelihood and severity of dementia was the number of concurrent pathologies ^78^. This is not a trivial undertaking, as the presence of any given condition—be it vascular disease or depression—likely influences the presentation of another condition such as dementia. Nonetheless, this is a major remaining issue that the field must soon tackle. One crucial thing to bear in mind is that nearly all aged human brains have some amount of vascular or parenchymal brain pathologies, some of which are often considered biomarkers for diseases such as AD. Thus, it is critical to come to understand which of these, where in the brain, and in what combinations constitute a serious threat to aging brain health.

## Summary

With the world’s aging population on the rise, it is imperative that we have a better understanding of the ‘healthy’ aging brain and how it differs from various pathological states. This is no easy feat. We suggest that in order to make inroads here, we must first focus on brain networks and systems that are selectively more vulnerable than others. We focused on episodic memory systems as memory complaints are quite common in the elderly and memory loss is one of the earliest hallmarks of AD. We first remind the reader that, absent neurodegenerative disease, aging is associated with synaptic dysfunction and not cell loss per se. With this in mind, we discuss the putative roles of inhibition and neurogenesis. We discuss regional vulnerability to neurodegenerative change, focusing on the EC but also extending to other recently identified subcortical targets. Finally, we discuss an important role for research considering individual differences and disease comorbidities in understanding neurocognitive changes in the aging brain. While by no means exhaustive, these are some of the important themes we think should guide research in the area over the coming few years.
